# Synthetic wavelength interferometry of an optical frequency comb for absolute distance measurement

**DOI:** 10.1038/s41598-018-22838-0

**Published:** 2018-03-12

**Authors:** Guanhao Wu, Lei Liao, Shilin Xiong, Guoyuan Li, Zhijian Cai, Zebin Zhu

**Affiliations:** 1 State Key Laboratory of Precision Measurement Technology and Instruments, Department of Precision Instrument, Tsinghua University, Beijing, 100084 China; 2 Satellite Surveying and Mapping Application Center, NASG, Beijing, 100048 China; 3 College of Physics, Optoelectronics and Energy, Soochow University, Suzhou, 215006 China

## Abstract

We present a synthetic-wavelength based heterodyne interferometer of optical frequency combs with wide consecutive measurement range for absolute distance measurement. The synthetic wavelength is derived from two wavelengths obtained by two band-pass filters. The interferometric phase of the synthetic wavelength is used as a marker for the pulse-to-pulse alignment, which greatly improves the accuracy of traditional peak finding method. The consecutive measurement range is enlarged by using long fiber to increase the path length difference of the reference and measurement arms. The length of the long fiber is stabilized according to the interferometric phase of a CW laser. The experimental results show the present system can realize an accuracy of 75 nm in 350 mm consecutive measurement range.

## Introduction

High accuracy distance measurement is essential for various applications such as industrial sensing of critical dimensions, and satellite formation in space missions^1^. Traditionally, single wavelength interferometry with fringe counting is employed for these applications. However, fringe counting is susceptible to cause loss of absolute position^2^. Multiple-wavelength interferometry can be used to realize absolute distance measurement avoiding fringe counting^3^. However, to realize large measurement range, a number of synthetic wavelengths are needed, which will result in a complicated system of multiple lasers or a low measurement speed due to wavelength scanning. Thanks to the advent of optical frequency comb which has led to revolutionary progress in absolute distance measurement^4^.

An optical frequency comb emits evenly spaced ultra-short pulse train with a broad spectrum consisting of discrete, uniform mode-spacing narrow lines. When its mode frequencies are referenced to a frequency standard, it becomes an ultra-accurate ruler in space, time and frequency domains^5^. Such inherent advantages are attractive for absolute distance measurement. Since the first work undertaken by Minoshima *et al*.^6^, various methods have been reported for such studies^7–22^, e.g., using the comb as a wavelength or frequency standard for CW lasers^7–11^, using a pair of frequency combs to realize time-of-flight measurement^12–18^, using the comb as a light source of dispersive interferometry^19–22^, and using the adjacent pulse repetition interval length (APRIL) as a scale^23–32^. Among the methods mentioned above, the APRIL is a simple and effective scheme based on an unbalanced Michelson interferometer^23,24^ which has been widely used in absolute distance measurement^25–32^. By adjusting the repetition rate (*f*_rep_) of the frequency comb to make the pulses from reference and measurement arms overlap, the target distance can be determined by accurate measurement of *f*_rep_^23^. Generally, two factors are extremely important to the performance of this method. The first is how to judge the pulses overlap, because the accuracy of pulse-to-pulse alignment determines the accuracy of distance measurement^32^. The second one is that limited tunable range of *f*_rep_ limits the consecutive measurement range, which leads to large ‘dead zone’ of this method^33^.

The accuracy of such distance measurement depends mainly on the knowledge of relative positions of the two overlapped pulses, i.e., pulse-to-pulse alignment. Conventionally, the peak position of interferogram envelope of the overlapped pulses is used for the pulse-to-pulse alignment^25–27^. Hereafter this method is referred to as peak finding method. It can realize micrometer or even sub-micrometer level alignment since the pulse width can be very narrow, but the accuracy is affected by the intensity noise of the interferogram envelope. Moreover, such accuracy is still not enough for ultra-precision applications. In order to improve the accuracy of pulse-to-pulse alignment, several attempts have been made on improving the peak finding method^30,31^ or linking the peak finding with the interferometric phase^32^. In our previous study, we proposed a two-color heterodyne interferometry based on the fundamental and second harmonic (SH) of optical frequency combs^32^. The synthetic wavelength derived from the virtual second harmonic (half wavelength of the fundamental) and the real second harmonic (generated by nonlinear crystal) was used to bridge the gap between peak finding method and the single-wavelength heterodyne interferometry. However, the system that requires a fully stabilized comb, second harmonic generation and two acousto-optic modulators is complicated for practical applications.

When applying the APRIL method, the consecutive measurement range depends on the tunable range of the cavity length and the optical path length difference (OPLD) between the measurement and reference arms^33^. For example, we denote the optical length of the cavity (equal to the optical distance between adjacent pulses) as *L*_pp_, and denote its tuable range as Δ*L*_max_. Assuming that OPLD is *k* times of *L*_pp_, it can realize a consecutive measurement range of *k*Δ*L*_max_/2 (consider the round trip of the beam) for distance measurement. Consequently, if *k* > *L*_pp_/Δ*L*_max_, the consecutive measurement range is large enough that no ‘dead zone’ exist. When we measure a short distance, we can add additional length delay in the referenc/measurement arm to increase OPLD to realize ‘multiplication effect’ to remove the ‘dead zone’. This concept has been applied in surface profilometry^34^ and optical sampling by cavity tuning^35,36^. It is also promising to be combined with the APRIL method.

In the present study, we present a simplified synthetic-wavelength interferometry of optical frequency combs for absolute distance measurement. The synthetic wavelength is derived from two wavelengths obtained by two band-pass filters. The interferometric phase of the synthetic wavelength is used as a marker for the pulse-to-pulse alignment. Thus, the carrier-envelope-offset frequency (*f*_ceo_) is not sensitive to the measurement results in this system^37^. In order to extend the consecutive measurement range, we employ long fiber to increase the OPLD of the reference and measurement arms. Consequently, the present system can realize large consecutive range distance measurement with high accuracy.

## The Measurement Principle

### Experimental setup

**Figure 1:**
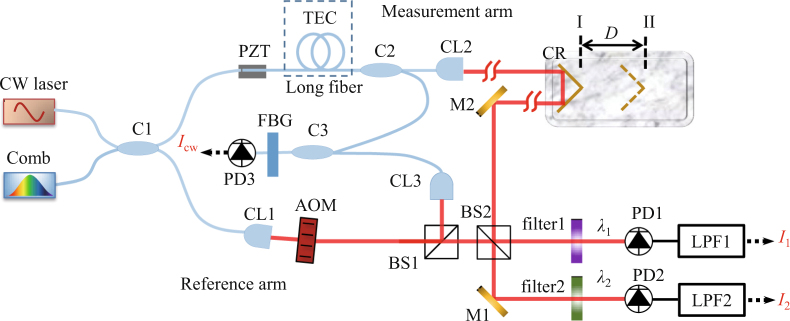
Schematic of experimental setup. Blue lines: fiber or fiber devices; red lines: optical paths in free space. C1–3: Fiber Coupler; CL1–3: Collimator lens. BS1–2: Beam splitter; M1–2: Mirror; CR: Corner reflector; PD1–3: Photodetector; LPF1–2: low-pass-filter; FBG: fiber Bragg grating filter. I and II indicate two positions of CR.

A home-made mode-locked Er:fiber ring laser is used as the frequency comb source (Fig. 1). Its optical power is 8 mW. The *f*_rep_ is stabilized referencing to a rubidium atomic clock (SIM940, Stanford Research Systems) and *f*_ceo_ is free running. The *f*_rep_ of the comb is 75.0 MHz, and it can be varied coarsely up to 1.9 MHz by a fiber delay line and finely up to 38 Hz by PZT-driven fiber stretching. The corresponding optical distance between adjacent pulses is *L*_pp_ = 4 m, and it can be varied up to Δ*L*_max_ = 100 mm.

The pulse train from the comb is sent to a coupler (C1) and divided into the reference and measurement arms. In the reference arm, an acousto-optic modulator (AOM) shifts the optical frequency by 80 MHz. In the measurement arm, there is long fiber (51 m) composed of PM fiber and dispersion compensation fiber. A corner reflector (CR) is placed on a translation stage and used as the target mirror, whose displacement is monitored by a commercial heterodyne interferometer (PT-1105c, Pretios Inc., not shown in this figure). The OPLD between the reference and measurement arms equal to 17 *L*_pp_. Thus, the consecutive measurement range of this system is 8.5Δ*L*_max_ = 850 mm. The pulses from the two arms overlap at a beam splitter (BS2). The interference signals are filtered by two band-pass filters (filter1, center wavelength *λ*_1_ = 1559 nm, bandwidth 7 nm; filter2, center wavelength *λ*_2_ = 1567 nm, bandwidth 7 nm) and detected by two Photodetectors (PD1 and PD2), respectively. In order to cancel out the drift and phase noise caused by the long fiber, a CW laser (center wavelength 1550 nm, Koheras Basik E15, NKT Photonics) which is locked to another fully stabilized comb is used to build another heterodyne interferometer. This interferometer shares the reference arm and the long fiber in measurement arm with the interferometer of the comb. The interference signal *I*_cw_ is detected by PD3. Before PD3, a 100 GHz (∼0.8 nm) fiber Bragg grating (FBG) band-pass filter (center wavelength 1550 nm) is used for improving the signal-to-noise of *I*_cw_. The phase of *I*_cw_ is used for the feedback control of the length of long fiber. We use temperature control (TEC) of the long fiber to compensate its drift and use a Piezoelectric Transducer (PZT) to stretch the fiber for fast control.

In the reference arm, the AOM shifts the optical frequency by *f*_Δ_ = 80 MHz. The *m*^th^ mode of the comb in reference arm can be expressed by:

1 $${f}_{{\rm{ref}}}(m)=m{f}_{{\rm{rep}}}+{f}_{{\rm{ceo}}}+{f}_{\Delta }.$$

The *m*^th^ mode of the comb in the measurement arm can be expressed by:

2 $${f}_{{\rm{mea}}}(m)=m{f}_{{\rm{rep}}}+{f}_{{\rm{ceo}}}.$$

Therefore, when the pulses from the reference arm and the measurement arm overlap, the frequency of beat signal can be expressed by *f*_Δ_ − *N . f*_rep_ = (80 − *N* 75) MHz (*N* is an integer). We use low-pass-filters (LPF1 and LPF2) after PD1 and PD2 to select the lowest-frequency beat signal (*f*_Δ_ − *f*_rep_ = 5 MHz) as the heterodyne signals (*I*_1_ and *I*_2_). The phase of the heterodyne interference signals from PD1 and PD2 (denoted as *ϕ*_1_ and *ϕ*_2_) is measured by a lock-in amplifier (HF2, Zurich Instruments). The heterodyne interference signal of the CW laser equals to *f*_Δ_ = 80 MHz. Its phase (*ϕ*_cw_) is measured by another lock-in amplifier (SR844, Stanford Research Systems). *ϕ*_cw_ is stabilized by feedback control of length of long fiber.

### Measurement principle

During the measurement, the target mirror was moved from position I to position II by a distance *D*. At both positions, peak finding method was applied for *I*_1_, i.e., the intensity of *I*_1_ was maximized by adjusting *f*_rep_. Here, it is not necessary to maximize the intensity precisely, because we will use the interferometric phase of the synthetic wavelength as a marker for the pulse-to-pulse alignment. The synthetic wavelength is in the magnitude of hundreds of micrometers, so it does not require very precise pre-alignment of the pulses.

**Figure 2:**
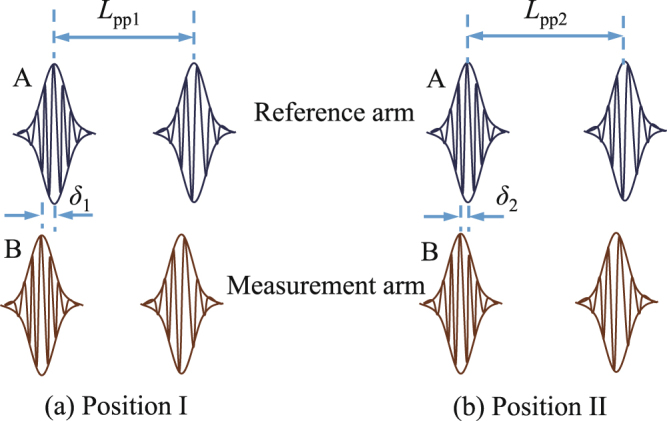
Demonstration of the pulses overlap from (**a**) position I to (**b**) position II.

After applying peak finding method for *I*_1_ at position I, we assume pulse A of reference arm overlaps with pulse B of measurement arm. Normally, pulses A and B cannot overlap perfectly due to the accuracy limitation of peak finding method. The distance between the peaks of the two pulses is denoted as *δ*_1_ (Fig. 2a). When the target mirror CR was moved by *D* to position II, resulting a relative displacement of *D* between the pulses A and B. In case of *D* < *L*_pp_/2*n*_g_ (*n*_g_ is the group refractive index of air), applying peak finding method at position II will compensate this relative displacement back to make the two pulses overlap again with a peak distance of *δ*_2_ (Fig. 2b). From position I to position II, we denote the optical distance between adjacent pulses change from *L*_pp1_ to *L*_pp2_, and the change value is ${\rm{\Delta }}{L}_{{\rm{pp}}}={L}_{{\rm{pp2}}}-{L}_{{\rm{pp1}}}=c/{f}_{{\rm{rep2}}}-c/{f}_{{\rm{rep1}}}$ (*c* is the speed of light in vacuum, *f*_rep1_ and *f*_rep2_ are the repetition rate corresponding to positions I and II). We also record the interferometric phase change as Δ*ϕ*_1_ and Δ*ϕ*_2_ for the two wavelengths. The relation between target distance *D* and the parameters recorded can be given by:

3 $${\delta }_{2}-{\delta }_{1}=2D-17{\rm{\Delta }}{L}_{{\rm{pp}}}/{n}_{{\rm{g}}}=({N}_{1}+{\rm{\Delta }}{\varphi }_{1}/2\pi ){\lambda }_{1},$$

4 $${\delta }_{2}-{\delta }_{1}=2D-17{\rm{\Delta }}{L}_{{\rm{pp}}}/{n}_{{\rm{g}}}=({N}_{2}+{\rm{\Delta }}{\varphi }_{2}/2\pi ){\lambda }_{2},$$

where *N*_1_ and *N*_2_ are the fringe orders to be determined. Note that the difference of the refractive index of air between the two wavelengths are ignored. It is necessary to point out the phase of the heterodyne signal are the integration of phases from all comb modes within the pass band, it corresponds to an effective center wavelength of the heterodyne interference signal. Here, we use the center wavelength of the filters as the effective center wavelength. More details about how to calculate the effective center wavelength can be found in the supplementary file.

From Eqs. (3) and (4), we can derive that

5 $${\delta }_{2}-{\delta }_{1}=2D-17{\rm{\Delta }}{L}_{{\rm{pp}}}/{n}_{{\rm{g}}}=({N}_{{\rm{s}}}+{\rm{\Delta }}{\varphi }_{{\rm{s}}}/2\pi ){\lambda }_{{\rm{s}}},$$

where ${\lambda }_{s}={\lambda }_{2}{\lambda }_{1}/({\lambda }_{2}-{\lambda }_{1})=305.4\,{\rm{\mu }}m$ is the synthetic wavelength, the corresponding phase ${\rm{\Delta }}{\varphi }_{s}={\rm{\Delta }}{\varphi }_{2}-{\rm{\Delta }}{\varphi }_{1}$ and *N*_s_ = *N*_2_ − *N*_1_. Note that the peaking finding method can easily ensure *δ*_1_ and *δ*_2_ be smaller than several micrometers, which is much smaller than *λ*_s_. Thus, *N*_s_ = 0. Therefore, we can obtain the target distance *D* according to Eq. (5) and the parameters recorded at positions I and II.

**Figure 3:**
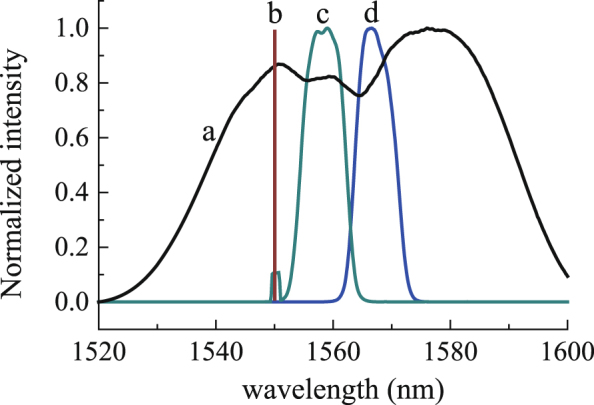
Optical spectra of the interference signals. (a) The optical spectrum of the comb; (b) indicates the wavelength of the CW laser; (c) the optical spectrum after filter1, there is a small bump at 1550 nm which indicates the spectrum of the CW laser is not suppressed perfectly; (d) the optical spectrum after filter2.

The optical spectrum of the comb is wide, and its full width at half maximum (FWHM) is more than 50 nm (Fig. 3a). Hence, there is wide space for selecting the center wavelengths (*λ*_1_ and *λ*_2_) of filters 1 and 2. To obtain an appropriate synthetic wavelength, we should pay attention to the following three points. (1) The synthetic wavelength should be long enough to avoid unambiguity of *N*_s_ in Eq. (5). (2) Based on point 1, shorter synthetic wavelength is favorable to obtain higher accuracy in distance measurement because the accuracy in phase measurement is fixed. (3) The transmission spectra of filters 1 and 2 should not overlap with that of the CW laser to avoid crosstalk. In the present system, the wavelength of the CW laser is 1550 nm, the transmission spectra of the filters are shown in Fig. 3. Please note that even under the three conditions mentioned above, there are a lot choices of the combination of *λ*_1_ and *λ*_2_. We did not make specific optimization in selecting *λ*_1_ and *λ*_2_ for the present system.

## Results

### Evaluation of long fiber stabilization

**Figure 4:**
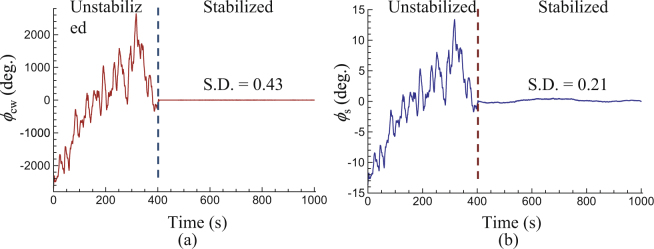
Performance of long fiber stabilization. (**a**) Interferometric phase (*ϕ*_cw_) of the CW laser. (**b**) Interferometric phase (*ϕ*_s_) of the synthetic wavelength.

In the present system, we used long fiber to increase the OPLD between the measurement and reference arms to extend the consecutive measurement range. The drift or phase noise of the long fiber affects the interferometric phase measurement directly. Therefore, we stabilized the long fiber according to the interferometric phase (*ϕ*_cw_) of the CW laser during the distance measurement. Figure 4 shows the stabilization results. In the first 400 s, the long fiber is not stabilized, *ϕ*_cw_ drifts nearly 5 thousands degrees (Fig. 4a) and interferometric phase (*ϕ*_s_) of the synthetic wavelength drifts nearly 26 degrees (Fig. 4b), corresponding to nearly 20 μm in optical length change.

When the long fiber is stabilized, *ϕ*_cw_ becomes very stable. It shows a standard deviation (S.D.) of 0.43 degree in 600 s (Fig. 4a), corresponding to 1.8 nm in optical length. In comparison, *ϕ*_s_ is not so flat when the long fiber is stabilized. It shows a S.D. of 0.21 degree in 600 s (Fig. 4b), corresponding to 180 nm in optical length. This is reasonable, because the interferometers of the CW laser and the comb are not in total common path. For example, the position of the target mirror CR may have some drift during the stabilization period of 600 s, and it will cause *ϕ*_s_ change. The residual instability of *ϕ*_s_ can be improved by decreasing the non-common path of the two interferometers and improving the environmental stabilities. Altogether, the long fiber stabilization decreases the interferometric phase drift greatly, which can ensure high-accuracy distance measurement of the present system.

### Results of absolute distance measurement

To evaluate the measurement accuracy of this system, we test the repeatability of the system firstly. The target mirror CR was moved by 100 mm, and the accurate displacement is given by a commercial heterodyne interferometer. The difference between the results obtained from the present method and the commercial interferometer indicates the measurement error (*ε*). We repeated the measurement 8 times with a time interval of 10 minutes. The measurement errors (*ε*) ranged from −83 nm to 92 nm with a standard deviation of 71 nm.

**Figure 5:**
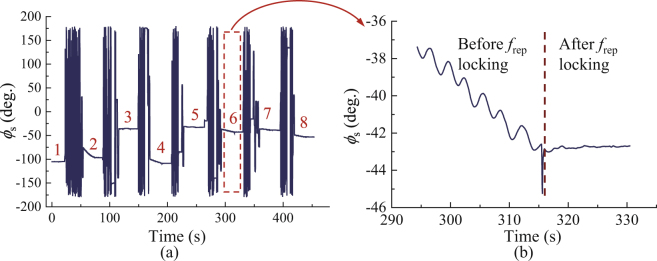
Interferometric phase (*ϕ*_s_) of the synthetic wavelength recorded in the distance measurement. (**a**) *ϕ*_s_ recorded in the 7-step movement, corresponding to 8 positions. For each step measurement, it only costs tens of seconds. (**b**) Expanded view of *ϕ*_s_ at position 6. We only use the *ϕ*_s_ after repetition rate locking for distance calculation.

Secondly, we made a linear measurement comparison with a commercial heterodyne interferometer. The target mirror CR was moved by 7 steps at an increment of 50 mm. The whole experiment was accomplished in 450 s and *ϕ*_s_ was recorded during the experiment (Fig. 5). We measured *ϕ*_s_ at 8 positions in total (Fig. 5a). Once the target mirror moved, we should scan the repetition rate to make the pulses overlap again. Thus, *ϕ*_s_ changes randomly between two adjacent positions, which indicates the repetition rate scanning. Please note it is not necessary to record *ϕ*_s_ during repetition rate scanning, we only need to know *ϕ*_s_ at different positions and then we can obtain the absolute distance between the two positions. At each position, we should lock the repetition rate after the repetition rate scanning process (Fig. 5b). After that, *ϕ*_s_ became stable and it was recorded for nearly 10 s. The average value during the 10-s recording was used for distance calculation, and its standard deviation is about 0.1°, corresponding to 42 nm in distance. During the measurement, the temperature, humidity and air pressure were recorded for calculation of the refractive index of air.

**Figure 6:**
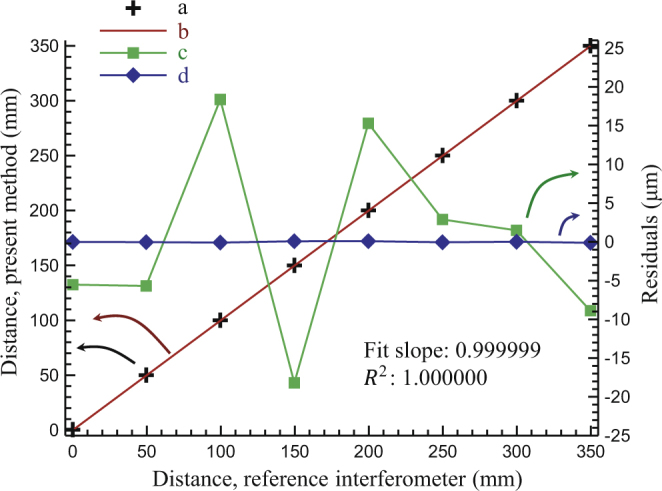
Experimental results versus the data obtained by the reference interferometer. (**a**) Distance data measured, (**b**) linear fitting of the data measured, (**c**) residuals of peak-finding method, (**d**) residuals of final results. a and b refer to the left longitudinal coordinates; c and d refer to the right one. They are indicated by the arrows.

We made a comparison of the distances measured by using the present method and the results of the commercial interferometer (Fig. 6). The final distance measurement results (Fig. 6a) are in good accordance with the results of the reference interferometer. By applying linear fitting (Fig. 6b), the slope is 0.999999 and the correlation coefficient (*R*^2^) is 1.000000. The residuals are ranged from −123 nm to 94 nm with a standard deviation of 75 nm (Fig. 6d). To demonstrate the advantages of present method, the residuals of the measurements by only applying the peak finding method are also shown (Fig. 6c) for comparison, which are ranged from −18 µm to 18 µm. Note that the accuracy of peak finding method here is lower than that in other reports^25,27^. In previous studies^25,27^, piezo is used for fine scanning to get the envelope of the cross-correlation function and obtain the intensity peak. In contrast, as mentioned in the section of measurement principle, it is not necessary to maximize the intensity precisely for this system. Because the synthetic wavelength is hundreds of micrometers, so it does not require very precise pre-alignment of the pulses. We only adjust the motor-driven fiber delay-line in the cavity, and make the intensity of the interference signal close to the peak value. Consequently, we can accomplish the peak finding process quickly but with a lower accuracy. Fortunately, this accuracy is enough to link with the synthetic wavelength interferometry.

## Discussion

In this experiment, we only demonstrated a consecutive measurement range of 350 mm. It is limited by the translation range of the stage. As analyzed in Section 2.1, the consecutive measurement range of the present system is 850 mm. It can be extended further by increasing the OPLD, e.g. increase the length of the long fiber. Once the consecutive measurement range reaches half *L*_pp_, the system can realize absolute distance measurement without ‘dead zone’.

It is necessary to point out *f*_ceo_ is free running during the experiment, which greatly decrease the complexity and cost of the laser source. Although *f*_ceo_ may drift during the measurement and cause some drift in *ϕ*_1_ and *ϕ*_2_, they are cancelled out in *ϕ*_s_ (*ϕ*_s_ = *ϕ*_2_ − *ϕ*_1_). Therefore, using the synthetic wavelength as a marker for the pulse-to-pulse alignment and distance measurement is promising for practical applications. Although we cannot improve the accuracy furtherly by employing the interferometric phase of a single wavelength^32^, the results that 350 mm consecutive measurement range with 75 nm accuracy (1*σ*) demonstrated here is enough for various applications. Note that we used a fully-stabilized comb as a reference for the CW laser. Actually, it is not necessary if we use a frequency-stabilized CW laser instead of the present CW laser.

Another advantage of this method is that the interferometric phase of the synthetic wavelength is used quantify a very small distance (e.g. *δ*_1_ and *δ*_2_ shown in Fig. 2) between the peaks of two overlapped pulses. No matter how long distance we measure, the fringe order (*N*_s_) of the synthetic wavelength is always zero. Thus, the accuracy of the synthetic wavelength itself is not a severe factor in the measurement since its error will not be accumulated by the fringe order. This feature is different from traditional fringe counting interferometry, and it is very attractive for long distance measurement.

## Conclusions

We proposed a synthetic-wavelength based heterodyne interferometer of optical frequency combs with wide consecutive measurement range for absolute distance measurement. The synthetic wavelength was derived from two wavelengths obtained by two band-pass filters, which simplified the complexity of synthetic wavelength generation. The interferometric phase of the synthetic wavelength was used as a marker for the pulse-to-pulse alignment, which greatly improved the accuracy of traditional peak finding method. Furthermore, the interferometric phase of the synthetic wavelength is immune to the drift of the carrier-envelope-offset frequency, which is attractive for practical applications. In order to extend the consecutive measurement range, we employed long fiber to increase the path length difference of the reference and measurement arms. The length of the long fiber was stabilized according to the interferometric phase of a CW laser. The repeatability measurement of 100 mm distance shows an accuracy better than 71 nm. By making a linear measurement comparison with a commercial interferometer, the present system showed an accuracy of 75 nm in 350 mm consecutive measurement range. This method can be widely used in various applications demanding high-accuracy distance measurement.

### Data Availability

The datasets generated during and/or analyzed during the current study are available from the corresponding author on reasonable request.

## Electronic supplementary material

Supplementary Information
