# Supplementary material for: Synthetic wavelength interferometry of an optical frequency comb for absolute distance measurement
Source: Sci Rep. 2018 Mar 12;8:4362. doi: 10.1038/s41598-018-22838-0 (PMC5847515; doi:10.1038/s41598-018-22838-0)
Supplement: Supplementary file 1 — Supplementary Information [file 41598_2018_22838_MOESM1_ESM.pdf]

SUPPLEMENTAL MATERIAL:

Synthetic wavelength interferometry of an optical frequency comb for absolute distance measurement

Guanhao Wu[1,*], Lei Liao[1], Shilin Xiong[1], Guoyuan Li,[2] Zhijian Cai,[3] and Zebin Zhu[1]

[1]*State Key Laboratory of Precision Measurement Technology and Instruments, Department of Precision Instrument, Tsinghua University, Beijing 100084, China*

[2]*Satellite Surveying and Mapping Application Center, NASG, Beijing, 100048, China*

[3]*College of Physics, Optoelectronics and Energy, Soochow University, 215006, China*

*[*guanhaowu@mail.tsinghua.edu.cn](mailto:guanhaowu@mail.tsinghua.edu.cn)*

Calculation of the effective center wavelength of the interference signal

The electric field of an optical frequency comb in vacuum can be expressed by:

$$E(t,x) = \sum_n A_n e^{i[2\pi f_n (t - x/c) + \varphi_n]} \tag{1}$$

where $f_n = nf_{rep}+f_{ceo}$ is optical frequency of nth mode, A_n is amplitude of nth mode, φ_n is initial phase of nth mode, t is time, x is the path length, and c is speed of light in vacuum.

We denote E_1 and E_2 as the electric fields of measurement arm and reference arm respectively. Note that E_2 has a frequency shift Δf by AOM. They can be expressed by:

$$E_1 = \sum_n A_n e^{i[2\pi f_n (t - x_1/c) + \varphi_n]}, \tag{2}$$

$$E_2 = \sum_n A_n e^{i[2\pi (f_n + \Delta f)(t - x_2/c) + \varphi_n]}, \tag{3}$$

where A_n and the total modes number n are determined by the spectrum of the band-pass filter used before the detector.

The interference signal is

$$I = \left|E_1 + E_2\right|^2 = (E_1 + E_2)\cdot(E_1 + E_2)^*. \tag{4}$$

The heterodyne interference signal (after low pass filter) is

$$I_{\mathrm{Het}} = \sum_n A_n^2 \cos[2\pi\Delta f (t - \frac{x_2}{c}) + 2\pi f_n \frac{x_1 - x_2}{c}]. \tag{5}$$

Please note the heterodyne beat frequency can be Δf or $(\Delta f – f_{rep})$. In Eq. (5), we use Δf for simplicity. I_{Het} is the sum of a series of cosine signals with a same frequency Δf, thus itself can be expressed as a simple cosine function with the same frequency Δf:

$$I_{\mathrm{Het}} = a\cos[2\pi\Delta f (t - \frac{x_2}{c}) + \alpha], \tag{6}$$

where a is the amplitude of I_{Het} and α is the phase of I_{Het}. According to Eqs. (5) and (6), we can deduce that

$$a\cos\alpha = \sum_n A_n^2 \cos 2\pi f_n \frac{x_1 - x_2}{c}, \tag{7}$$

$$a\sin\alpha = \sum_n A_n^2 \sin 2\pi f_n \frac{x_1 - x_2}{c}. \tag{8}$$

So that

$$\tan\alpha = \frac{\sum_n A_n^2 \sin 2\pi f_n \frac{x_1 - x_2}{c}}{\sum_n A_n^2 \cos 2\pi f_n \frac{x_1 - x_2}{c}}. \tag{9}$$

Using a numerical method, we can set a series of (x_1−x_2) and calculate a serices of α according to Eq. (9). Then we can calculate the effective center frequency (wavelength) of the interference signal.

In particular, if the optical spectrum of the band-pass filter is symmetric with the center frequency f_c, the calculation can simplified. We consider two mode lines symmetric with f_c, and modes orders are n_c-n_i and n_c+n_i, where n_c is the order of f_c. We have $A_{n_c - n_i} = A_{n_c + n_i}$, so the sum signal of the heterodyne interference from the two spectral lines is

$$\begin{aligned}
&\cos[2\pi\Delta f(t - \frac{x_2}{c}) + 2\pi f_{n_c - n_i} \frac{x_1 - x_2}{c}] + \cos[2\pi\Delta f(t - \frac{x_2}{c}) + 2\pi f_{n_c + n_i} \frac{x_1 - x_2}{c}] \\
&= \cos[2\pi\Delta f(t - \frac{x_2}{c}) + 2\pi(f_{n_c} - n_i f_{\text{rep}})\frac{x_1 - x_2}{c}] \\
&\quad + \cos[2\pi\Delta f(t - \frac{x_2}{c}) + 2\pi(f_{n_c} + n_i f_{\text{rep}})\frac{x_1 - x_2}{c}] \\
&= 2\cos(2\pi n_i f_{\text{rep}} \frac{x_1 - x_2}{c}) \cos[2\pi\Delta f(t - \frac{x_2}{c}) + 2\pi f_{n_c} \frac{x_1 - x_2}{c}]
\end{aligned} \tag{10}$$

Thus we have

$$I_{\text{Het}} = \cos[2\pi\Delta f(t - \frac{x_2}{c}) + 2\pi f_{n_c} \frac{x_1 - x_2}{c}] \sum_n A_n^2 \cos[2\pi(n - n_c) f_{\text{rep}} \frac{x_1 - x_2}{c}]. \tag{11}$$

We denote that

$$I_{\text{Het}} = b\cos[2\pi\Delta f(t - \frac{x_2}{c}) + 2\pi f_{n_c} \frac{x_1 - x_2}{c}], \tag{12}$$

where

$$b = \sum_n A_n^2 \cos[2\pi(n - n_c) f_{\text{rep}} \frac{x_1 - x_2}{c}]. \tag{13}$$

Comparing Eqs. (6) and (12), we can obtain that

$$\alpha = 2\pi f_{n_c} \frac{x_1 - x_2}{c} \tag{14}$$

Therefore, the integration of phases from all comb modes is equal to the phase of the center mode when the optical spectrum of the band-pass filter is symmetric with the center frequency f_c.

For the present experimental system, optical spectrum of the two band-pass filters are both nearly symmetric with their center frequencies. Thus, we use the center wavelengths of the filters as the effective center wavelength of the interference signal. They are measured by an OSA (Optical Spectrum Analyzer, AQ6370C, YOKOGAWA). We also use the numerical method according to Eq. (9) to verify the effective center wavelengths, and find the differences are both less than 0.03 nm, the corresponding difference in synthetic wavelength is about 87 nm. The results are shown as follows:

	λ_1 (nm)	λ_2 (nm)	λ_s (μm)
OSA	1558.919	1566.945	304.440
Numerical method	1558.947	1566.971	304.353

As discussed in the manuscript, no matter how long distance we measure, the fringe order (N_s) of the synthetic wavelength is always zero. Thus, the accuracy of the synthetic wavelength itself is not a severe factor in the measurement since its error will not be accumulated by the fringe order. In our experiment (Figure 5), the phase change of synthetic wavelength (ϕ_s) is less than 90 deg., thus the error caused by the λ_s is less than 87/2×(90/360) = 11 nm, which is negligible.
